# Master of Puppets: How Microbiota Drive the Nematoda Ecology and Evolution?

**DOI:** 10.1002/ece3.71549

**Published:** 2025-08-19

**Authors:** Víctor José Trejo‐Meléndez, Jorge Contreras‐Garduño

**Affiliations:** ^1^ Laboratorio de Ecología‐Evolutiva, Escuela Nacional de Estudios Superiores, Unidad Morelia Universidad Nacional Autónoma de México Morelia Michoacan Mexico; ^2^ Programa de Doctorado del Posgrado en Ciencias Biológicas Universidad Nacional Autónoma de México Mexico City Mexico

## Abstract

In recent decades, the microbiota has emerged as a key driver of biological functions in metazoans, and nematodes are no exception. Advances in genomic technologies have enabled detailed exploration of nematode–microbiota interactions, revealing compelling insights. However, much of our current understanding is derived from studies on the model organism *Caenorhabditis elegans* , where the microbiota's role in shaping host phenotypes and genotypes has been extensively characterized. These studies have uncovered the selective pressures influencing the function, structure, and assembly of the microbiota, highlighting the dynamic interplay between nematodes and their associated microbial communities. Despite these findings, the ecological and evolutionary implications of the microbiota in nematodes remain underappreciated. Emerging evidence indicates that the microbiota can modulate nematode life‐history traits and mediate trade‐offs among fitness components. Moreover, mechanisms such as horizontal gene transfer from bacteria have been shown to alter nematode phenotypes and genotypes, facilitating adaptation to novel or challenging environments. In this review, we integrate life‐history theory into the nematodes–microbiota interactions, offering a framework to identify the mechanisms driving phenotypic variation in nematodes. Understanding these processes is essential for uncovering the evolutionary and ecological bases of metazoan diversification, with the microbiota acting as a crucial source of phenotypic and genetic variability.

## Graphical abstract

The nematode microbiota can influence life‐history traits, thereby shaping the species' ecology and evolutionary trajectories.

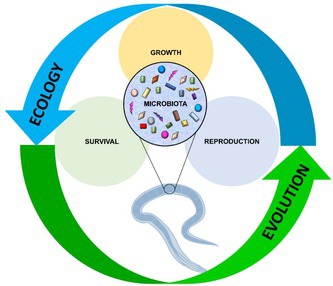

## 1 Introduction

The microbiota comprises functional communities of archaea, bacteria, viruses, anaerobic fungi, and protozoans that inhabit a wide range of metazoans (Berg et al. 2020; Moeller and Sanders 2020). With the advent of advanced metagenomic and metatranscriptomic techniques, the genetic repertoire and molecular products of these communities (collectively termed the microbiome) have been characterized across diverse eukaryotic hosts (Zilber‐Rosenberg and Rosenberg 2008). Among these microorganisms, bacteria have been the most extensively studied. Host‐associated bacterial communities are now recognized as major modulators of phenotype, influencing traits such as pathogen resistance (Kissoyan et al. 2022; Montalvo‐Katz et al. 2013), growth rate (Stuhr and Curran 2020), lifespan (Virk et al. 2012), and reproductive output (Haçariz et al. 2021). Furthermore, through mechanisms like horizontal gene transfer (HGT), microbiota can introduce genetic material to the host and modulate gene expression, thereby contributing to both phenotypic plasticity and genetic variation (Haegeman et al. 2011). Importantly, the microbiota is a dynamic system. Its composition and function can shift in response to internal host changes (e.g., diet, physiology) or environmental perturbations (Rosengaus et al. 2011). Such shifts can, in turn, lead to alterations in host phenotypes and genotypes, which become subject to natural selection (David et al. 2014; Gilbert et al. 2015). Despite growing recognition of its functional importance, the role of the microbiota in host ecology and evolution remains underexplored, representing a promising frontier for research. Integrating ecological and evolutionary perspectives into microbiome studies may provide key insights into phenotypic variation and adaptive processes in eukaryotes (Esser et al. 2019; Gould et al. 2018; Kolodny et al. 2020; Kolodny and Schulenburg 2020; Petersen et al. 2023; Suárez 2020). Invertebrate microbiotas, including those of nematodes, typically exhibit lower species richness than those of vertebrates (Douglas 2019), allowing more precise dissection of individual microbial functions within both the microbial community and the host. This has enabled experimental manipulation of bacterial combinations to evaluate host responses and cost–benefit trade‐offs over relatively short timescales. Nematodes are particularly valuable in this context due to their widespread use in a broad range of disciplines (including evolutionary biology, ecology, physiology, neurobiology, immunology, and genetics), with *Caenorhabditis elegans* serving as a central model organism (Backes et al. 2021; Dirksen et al. 2020). However, nematodes are recognized for living in diverse and complex microhabitats and for displaying wide lifestyles, such as free‐living or plant and vertebrate parasites (Parkinson et al. 2004). Additionally, many nematode species interact intimately with bacteria, using them as a food source, for environmental exploitation, for protection against pathogens, or to support metabolic processes essential for survival. In many cases, bacteria are integral to the nematode's natural history (Atibalentja and Noel 2008; Ott et al. 2004; Poinar Jr. and Hansen 1986; Setegn et al. 2024; Stock and Blair 2008; Topalović and Vestergård 2021). Studying nematode‐associated microbiotas thus offers a unique opportunity to uncover fundamental mechanisms underlying diversification within the phylum, particularly regarding the multiple, independent evolutionary origins of parasitism (Blaxter and Koutsovoulos 2015).

This review synthesizes current evidence on the nature of the microbiota and its role in the ecology and evolution of nematodes. We begin by highlighting well‐characterized symbioses between nematodes and bacteria, including roles in resistance to harsh marine conditions, facilitation of parasitism in plants, vertebrates, and insects, and bacterial pathogenesis within nematodes. Emphasis is placed on how microbial communities contribute to nematode fitness. Next, we examine the significance of *C. elegans* as a model organism, which has been instrumental in elucidating how microbiota composition, richness, and function are shaped by host genetics, bacterial gene products, and trophic interactions within the microbial community. We then outline the core functions of *C. elegans* microbiota and explore, from an eco‐evolutionary perspective, its impact on *C. elegans* life‐history traits, the resulting fitness trade‐offs, and adaptive responses to environmental variation. Finally, we provide a scope of the nematodes’ dismissed partners (or enemies): protozoans, fungi and viruses.

## 2 Recognized Bacteria–Nematode Complexes

**FIGURE 1:**
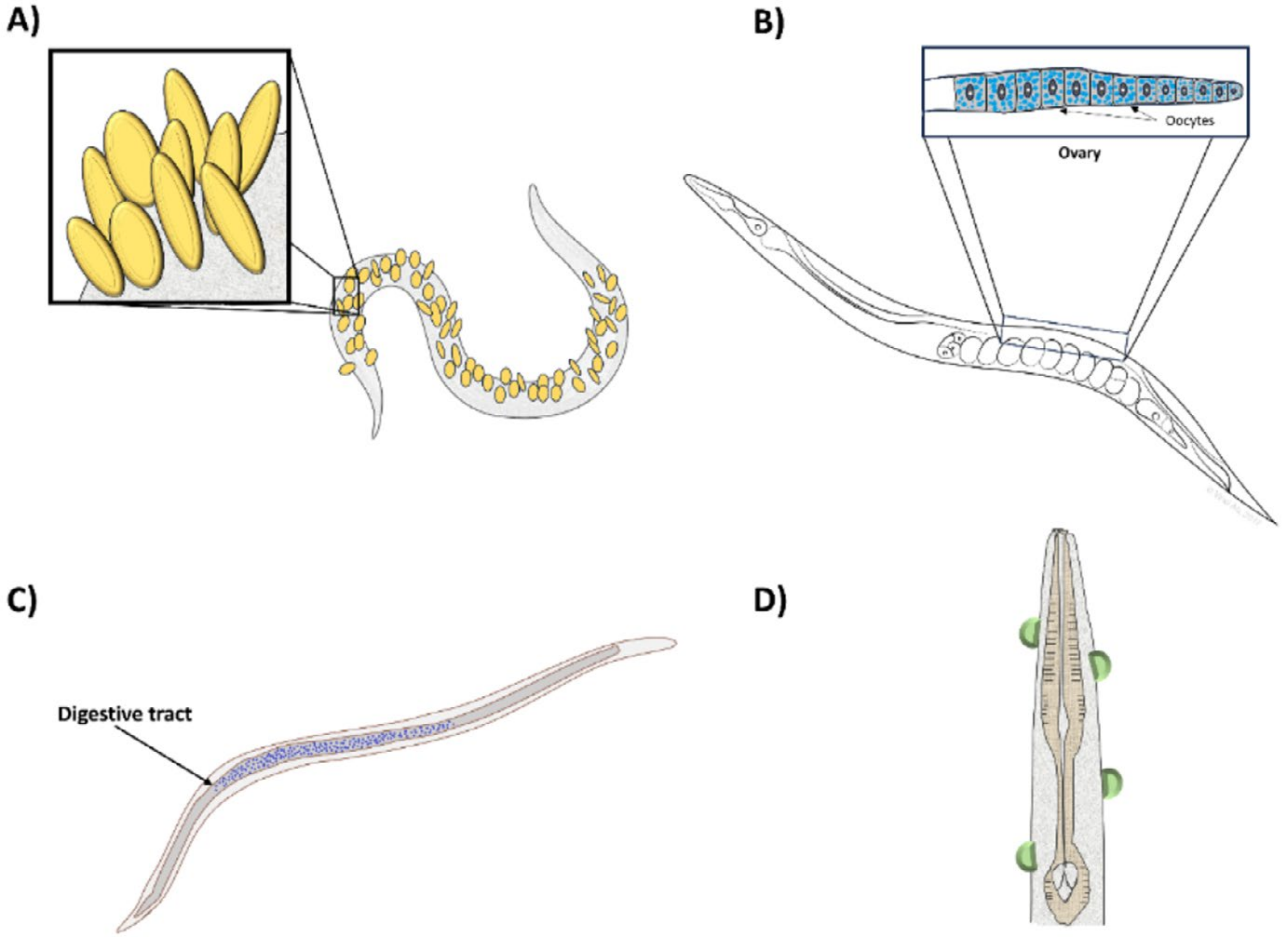
Schematic representation of recognized nematode–bacteria symbioses. (A) Ectosymbiosis: In free‐living marine nematodes, bacterial symbionts attach to the cuticle (yellow circles), forming a dense microbial coat that covers most of the nematodes body. In addition to this external association, marine nematodes also harbor internal microbiota along the digestive tract. (B) Intracellular endosymbiosis: Certain bacterial genera such as *Cardinium*, *Wolbachia*, and *Xiphinematobacter*, reside within nematode cells, occupying diverse regions including the gonads, intestinal lumen, and even intracellular vacuoles (blue dots). (C) Extracellular endosymbiosis: Unlike intracellular symbionts, these bacteria colonize internal tissues without invading host cells. Entomopathogenic nematodes such as *Heterorhabditis* host symbionts that line the intestinal tract (blue dots), whereas *Steinernema* species maintain their core symbionts within specialized intestinal vesicles. (D) Antagonistic ectosymbiosis: Pathogenic bacteria such as *Pasteuria* adhere to the nematode cuticle (green half‐circles), initiating a parasitic interaction that ultimately results in bacterial internalization and nematode death.

The interaction between nematodes and bacteria dates to the Paleozoic era (Poinar Jr. and Hansen 1986; Poinar 1983). Over millions of years, these interactions have evolved into diverse and intricate symbioses, spanning free‐living marine species, entomopathogens, and plant‐ and vertebrate‐parasitic nematodes. These associations encompass a continuum of relationships (from parasitic and pathogenic to mutualistic and commensal) that may be facultative or obligate, transient or persistent, and occur externally (ectosymbiosis) or internally (endosymbiosis; Figure 1). Given the extraordinary diversity within the phylum Nematoda, many such associations likely remain undiscovered. Bacterial partners can provide a range of benefits to their nematode hosts, from ecological advantages to vital physiological functions. Moreover, these interactions may shape nematode evolutionary trajectories through mechanisms such as host–microbe co‐evolution, including dynamics predicted by the Red Queen hypothesis (Davies et al. 2023; Morran et al. 2011). While early studies often focused on identifying a single “core symbiont,” they frequently overlooked the broader microbial community. Recent advances in molecular techniques now allow for genomic‐level analyses of nematode‐associated microbiota, revealing their significant roles in shaping nematode biology. In the next section, we explore the diversity of nematode‐bacteria associations and examine how microbial partners influence nematode ecology and evolution.

### 2.1 Marine Free‐Living Nematodes and Chemosynthetic Bacteria

Within the meiofauna, marine free‐living nematodes are notable for their symbioses with chemolithoautotrophic sulfur‐oxidizing bacteria (Ott et al. 2004). To date, such associations have been documented in species from three taxonomically disparate subfamilies: Stilbonematinae (Desmodorida: Desmodoridae), Astomonematinae (Monhysterida: Siphonolaimidae), and Oncholaiminae (Enoplida: Oncholaimidae; Bellec et al. 2019, 2020; Hentschel et al. 1999; Ott et al. 1991; Schuelke et al. 2018). These nematodes inhabit sheltered intertidal and subtidal sediments, often concentrating at the redox interface between oxygenated surface layers and deeper, anoxic zones. In these sulfur‐ and methane‐rich environments (typically hostile to most metazoans due to conditions like sulfide toxicity and oxidative stress) nematodes persist, in part due to their bacterial symbionts. For example, sulfide inhibits cytochrome c oxidase, while reactive oxygen species (ROS) can impair growth (Somero et al. 1989; Vismann 1991). Chemosynthetic symbionts appear to mitigate these effects, as demonstrated in species of Stilbonematinae and Oncholaiminae. In both groups, ectosymbiotic bacteria (primarily Campylobacterota and Gammaproteobacteria) are attached to the nematode cuticle (Bellec et al. 2018, 2019; Hentschel et al. 1999; Ott et al. 1991). Stilbonematinae species are characterized by species‐specific, highly structured bacterial coats: (1) multilayered coccoid bacteria in *Stilbonema* and *Leptonemella* (Polz et al. 1992); (2) monolayers of rod‐shaped bacteria in *Laxus*, *Robbea*, and *Catanema* (Polz et al. 1992); and (3) a filamentous bacterial layer up to 100 μm thick in *Eubostrichus* (Berger et al. 1996). In comparison, Oncholaiminae species such as *Metoncholaimus albidus* and *Oncholaimus dyvae* host monolayers of filamentous and/or rod‐shaped bacteria (Bellec et al. 2018, 2019). These nematodes exhibit vertical migrations between oxygen‐rich surface layers and sulfide‐rich sediments, enabling their symbionts to access both electron donors (e.g., hydrogen sulfide) and electron acceptors (e.g., oxygen; Hentschel et al. 1999). While the bacteria generate organic compounds through sulfur oxidation (Ott et al. 1991), their exact functional role remains unclear. Evidence suggests the bacteria serve as a mobile food source and assist the host in navigating between oxic and anoxic zones. Additional proposed functions include protection from sulfide toxicity (Hentschel et al. 1999; Ott et al. 2004, 1991) and contributions to denitrification (Petersen 2017). In contrast, Astomonematinae species such as *Astomonema* and *Parastomonema* harbor intracellular endosymbionts (Giere et al. 1995). These nematodes lack a functional mouth, possess a reduced intestine, and have a vestigial pharynx, indicating that oral feeding is absent (Ingels et al. 2023). Nonetheless, the detection of the *aprA* gene (adenosine‐5′‐phosphosulfate reductase), a marker of sulfur oxidation in their endosymbionts (Bellec et al. 2019; Musat et al. 2007), supports a nutritional role similar to that in stilbonematines. These findings suggest an obligate mutualism. However, unlike Stilbonematinae, the ecological roles of these endosymbionts remain poorly understood. Notably, it is unclear how astomonematines tolerate sulfide toxicity in the absence of the “bacterial shield” present in their ectosymbiotic relatives.

Studies characterizing the microbiota of marine nematodes remain limited. However, recent analyses of *Oncholaimus* species have revealed associations with diverse chemosynthetic bacterial lineages (Bellec et al. 2020). These include sulfur‐oxidizing bacteria such as *Sulfurimonas*, members of the *Thiotrichaceae*, and *Thermodesulfovibrio*, as well as sulfate‐reducing bacteria like *Desulfobacterales*. Methanotrophic taxa, including *Methylococcales*, *Zetaproteobacteria*, and *Verrucomicrobia*, were also identified. Additionally, lineages such as *Burkholderia* and *Pseudomonas* may contribute to metal chelation and pollutant degradation (Bellec et al. 2020; Lüddeke et al. 2012). These associations with chemosynthetic and detoxifying microbes are thought to play a key role in enabling nematodes to colonize and persist in the dynamic and often extreme conditions of the ocean floor. More recently, microbiota profiles have been described for bacterivore/detritivore marine nematodes that feed on fresh or decomposing macroalgae (Vafeiadou et al. 2022). These nematodes represent a cryptic species complex, exhibiting diverse feeding strategies (e.g., selective bacterivory) and varying tolerances to abiotic factors such as salinity and temperature (De Meester et al. 2011, 2016; Monteiro et al. 2018). Their microbiota includes a broad array of bacterial taxa (*Proteobacteria*, *Actinobacteria*, *Firmicutes*, and *Cyanobacteria*) as well as archaeal genera such as *Candidatus Nitrospumilus*, *Methanobacterium*, and *Methanobrevibacter* (Vafeiadou et al. 2022). The study suggests that microbiota composition contributes to differential resource exploitation and environmental resilience, facilitating ecological niche differentiation and promoting species coexistence through niche partitioning. Symbiotic associations between marine nematodes and chemosynthetic bacteria may similarly reflect such ecological specialization. In particular, the co‐occurrence of Stilbonematinae and Astomonematinae in similar habitats is thought to result from convergent evolution driven by symbiont‐mediated adaptation (Semprucci et al. 2025). Host factors, such as cuticle receptors or gut lumen properties, likely influence which bacterial species are able to attach and proliferate (Ott et al. 2004). Natural selection is expected to favor host individuals expressing receptors that promote associations with fitness‐enhancing symbionts. Understanding how marine nematodes adapt to diverse microhabitats through microbial associations is essential for uncovering the evolutionary dynamics of mutualism. Further research is needed to elucidate how nematodes regulate their microbiota, the nature of cooperation and competition within microbial communities, and the roles that microbiota play in nutrition, detoxification, immunity, and other key biological processes.

### 2.2 Plant Parasitic Nematodes Endosymbionts

Phytoparasitic nematodes are of global concern due to their substantial negative impact on agriculture (Singh et al. 2013). Recent studies have identified a range of bacterial endosymbionts in several plant‐parasitic nematode taxa (Brown 2018). These endosymbionts are largely restricted to three bacterial genera, each associated with specific nematode hosts: (1) *Cardinium* in cyst nematodes (*Globodera* spp., *Heterodera* spp.) and lesion nematodes (*Pratylenchus* spp.); (2) *Xiphinematobacter* in dagger nematodes (*Xiphinema* spp.); and (3) *Wolbachia* in both lesion (*Pratylenchus* spp.) and burrowing (*Radopholus* spp.) nematodes (Brown et al. 2018). *Cardinium* (phylum Bacteroidetes) is an intracellular endosymbiont localized in the gut, hypodermis, and sperm of *Globodera*, *Heterodera*, and *Pratylenchus* species (Brown 2018; Denver et al. 2016; Noel and Atibalentja 2006; Shepherd et al. 1973; Yang et al. 2017). It is also found in ovarian cells, the oviduct wall, and oocytes, consistent with vertical transmission (Brown 2018). Genomic analyses reveal that *Cardinium* lacks key biosynthetic pathways but is enriched in genes encoding transporters for oligopeptides and amino acids, suggesting dependence on the host for metabolite acquisition and energy (e.g., ATP). Notably, it retains complete biosynthetic pathways for biotin and lipoate.

The presence of numerous genes involved in host interaction and cellular regulation suggests a functional role for *Cardinium* in nematode physiology. For instance, genes associated with lipid storage, an antifeeding prophage (AFP)‐like protein secretion system, and ATP transport imply a role in fatty acid metabolism. This is supported by experimental evidence showing that *Cardinium* affects lipid metabolism in infective juveniles (Walsh et al. 1983). Many of these genes appear to have been acquired via HGT. HGT is widely recognized as a key mechanism in the evolution of plant parasitism in nematodes, with Bacteroidetes frequently serving as gene donors (Craig et al. 2009; Danchin et al. 2016; Haegeman et al. 2011; Jones et al. 2005; Rybarczyk‐Mydłowska et al. 2012). The acquisition of bacterial genes encoding cell wall‐degrading enzymes such as endoglucanases has enabled nematodes to exploit plant tissues, while vitamin biosynthesis genes (e.g., for B1 and B6) have enhanced their nutritional capabilities (Craig et al. 2009; Danchin et al. 2016). Remarkably, the cyst nematode *Heterodera glycines* harbors a bacterial‐like biotin synthase gene (*HgBioB*), which appears to contribute to virulence (Bekal et al. 2015), suggesting multifunctional roles for horizontally acquired genes in nematode adaptation and parasitism.

Dagger nematodes (*Xiphinema* spp.) are migratory ectoparasites that use a highly specialized odontostyle (stylet) to feed on the phloem of various plant roots (Baiai and Jairaipuri 1979; van den Berg et al. 2017). In addition to their direct impact on host plants, they act as vectors for economically significant nepoviruses, including *Cherry rasp leaf virus*, *Tobacco ringspot virus*, and *Tomato ringspot virus*, all of which contribute to substantial agricultural losses (Brown et al. 1993).

A notable feature of *Xiphinema* spp. is their association with *Xiphinematobacter*, an intracellular endosymbiont belonging to the phylum Verrucomicrobia. This bacterium is primarily located in the gut lumen of juveniles and adult females, with less frequent presence in males, and is also consistently found within oocytes, indicating vertical transmission (Coomans et al. 2000). Transmission electron microscopy (TEM) studies have shown that *Xiphinematobacter* species possess a thin peptidoglycan layer, a structural trait likely critical for symbiont survival by minimizing host immune responses (Otten et al. 2018; Palomares‐Rius et al. 2021). In addition, *Xiphinematobacter* is also implicated in inducing thelytokous parthenogenesis (Coomans and Claeys 1998; Luc et al. 1998). The consistent presence of the symbiont in reproductive tissues, along with strong cophylogenetic congruence between host and symbiont lineages, supports a long‐term, co‐evolved, and obligate mutualistic relationship (Mobasseri et al. 2018). Genomic analyses of *Xiphinematobacter* reveal a highly reduced genome lacking genes essential for a free‐living lifestyle, such as those involved in the biosynthesis of nonessential amino acids and many cofactors (Brown et al. 2015). In contrast, retained genes are enriched for pathways that complement host nutrition, including the biosynthesis of essential amino acids, vitamins, and cofactors. The genome also encodes pathways for lipoate synthesis (shared with *Cardinium*) and heme biosynthesis, a feature similarly observed in *Wolbachia* (Brown et al. 2015). Given that *Xiphinema* spp. feed on phloem sap, which is deficient in many essential nutrients, the functional capacities of *Xiphinematobacter* strongly suggest its role in supplementing the nematode's diet, further underscoring the obligate nature of the symbiosis (Brown et al. 2015, 2018). Notably, some *Xiphinema* species that do not rely on phloem feeding lack *Xiphinematobacter* endosymbionts, suggesting that either alternative nutritional strategies or symbionts may be involved (Brown et al. 2015). Indeed, limited studies on the microbiota of *Xiphinema* have revealed additional symbiotic candidates. For example, a member of the Betaproteobacteria, provisionally named *Xiphinematincola* sp., has been found in both the gut lumen and oocytes of *Xiphinema* (Palomares‐Rius et al. 2021). While the functional role of *Xiphinematincola* remains unclear, its localization pattern suggests it may provide complementary or redundant functions to those of *Xiphinematobacter*. Some researchers have proposed that physical limitations of the odontostyle might restrict bacterial entry into the gut, arguing against environmental acquisition of symbionts (Palomares‐Rius et al. 2021). However, alternative entry routes (such as through the vulva or anus) could enable microbial colonization. Supporting this, metagenomic analyses of *Xiphinema elongatum* have identified associated bacteria including *Sphingomonas*, *Clavibacter*, *Succinivibrio* sp., *Faecalibacterium prausnitzii* , and *Propionibacterium acnes* (Shokoohi and Masoko 2024). While metabolic functions of these taxa are known in other systems, their ecological or physiological roles within *Xiphinema* remain largely unexplored.

Microbiota associated with plant‐parasitic nematodes have been characterized in several species, including *Heterodera* spp., *Meloidogyne* spp., and *Pratylenchus* spp. (Cao et al. 2015; Elhady et al. 2017; Nour et al. 2003). Most studies to date have focused more on external (epibiotic) microbiota than on internal symbionts. However, investigations into the internal microbiota of *Meloidogyne incognita* revealed associations with members of *Alphaproteobacteria* and *Betaproteobacteria*, including taxa known for phytopathogenicity, nitrogen fixation, and nematode pathogenicity (Cao et al. 2015). Among the identified “core taxa” were *Pseudomonas*, *Rhizobium*, and *Acidovorax*. Interestingly, *Xiphinematobacter* was also detected in *M. incognita* , although only in the egg and juvenile stages. The presence of nitrogen‐fixing and cellulose‐degrading bacteria in the internal microbiota has led to hypotheses regarding their potential roles in nematode–plant interactions. Nitrogen‐fixing bacteria may aid in evading plant immune responses, as they are not typically recognized as harmful, potentially allowing nematodes to remain undetected (Cao et al. 2015; Topalović and Vestergård 2021). Conversely, cellulose‐degrading bacteria might facilitate root penetration and feeding (Tian et al. 2015; Yergaliyev et al. 2020). The external microbiota of *Meloidogyne* and *Pratylenchus* species is primarily composed of *Betaproteobacteria*, *Bacilli*, *Actinobacteria*, and some fungal taxa (Elhady et al. 2017; Nour et al. 2003). Comparisons between nematode‐attached and surrounding soil microbiota indicate selective microbial recruitment, likely mediated by the nematode's surface coat (a highly glycosylated lipoprotein layer known to interact with bacterial and fungal cells; Davies and Curtis 2011). This surface may promote the attachment of opportunistic microbes that contribute to plant infection (Elhady et al. 2017).

In *Heterodera glycines* , the cyst microbiota is enriched in Actinobacteria such as *Streptomyces*, *Agromyces*, and *Saccharothrix*, which are known for producing antimicrobial and cytotoxic compounds (Hashizume et al. 2001; Nam et al. 2001; Wright et al. 1998). These associations may provide protective benefits in the rhizosphere by suppressing microbial antagonists. Another striking example of symbiotic microbial function comes from *Bursaphelenchus xylophilus*, a nematode parasite of pine trees that feeds on parenchyma cells within woody tissue (Mota and Vieira 2008). In response to infection, host trees deploy chemical defenses such as terpenoids, which damage the nematode cuticle and impair development (Oku 1988). Transcriptomic analyses show that *B. xylophilus* lacks genes for terpenoid detoxification, including cytochrome P450 enzymes (Yan et al. 2012). However, metagenomic data reveal a diverse microbiota dominated by α‐, β‐, and γ‐Proteobacteria and, to a lesser extent, Bacteroidetes, which collectively encode enzymes for detoxifying xenobiotics and plant‐derived compounds such as benzoate and α‐pinene (Cheng et al. 2013; Kawazu et al. 1996). Collectively, these examples underscore the essential role of microbial associates in overcoming ecological constraints faced by phytoparasitic nematodes. These functions include assisting in plant exploitation, suppressing or evading plant defenses, and supplementing nutritional deficiencies. Such interactions may drive the evolution of specific symbioses and contribute to the diversification and ecological success of plant‐parasitic nematodes.

### 2.3 Vertebrate Parasites: Filarial Nematodes and the Endosymbiont Wolbachia

Filarial nematodes utilize mosquitoes as vectors to infect humans (Manoj et al. 2021). Mosquitoes acquire microfilariae from the bloodstream of an infected human during a blood meal. Within the mosquito, the nematodes develop into infective larvae, which subsequently migrate to the mosquito's mouthparts. During a subsequent bite, the larvae are transmitted to a new human host, where they mature and reproduce. In the human body, these parasites can trigger inflammation of the lymphatic system, leading to symptoms such as lymphadenitis, fever, and lymphedema. Chronic infections may progress to severe lymphatic damage, resulting in elephantiasis (Manoj et al. 2021; Sanaei et al. 2021). Nematode genera responsible for filariasis, including *Onchocerca*, *Wuchereria*, *Brugia*, and *Dirofilaria*, host a well‐characterized endosymbiont: *Wolbachia* (Bouchery et al. 2013; Haegeman et al. 2009; Setegn et al. 2024). These bacteria reside within host cell vacuoles and are particularly abundant in somatic tissues such as the lateral cords, as well as in the female reproductive system, especially the ovaries. *Wolbachia* is known for its diverse interactions with invertebrates, ranging from parasitism to mutualism in arthropods and a well‐established obligate mutualism in filarial nematodes (Voronin et al. 2012). Its contribution to nematode biology includes enhancing and complementing key metabolic pathways, which substantiates its essential role (Hosokawa et al. 2010; Nikoh et al. 2014). Numerous studies have demonstrated *Wolbachia*'s influence on nematode reproduction, development, and longevity (Darby et al. 2012; Fenn and Blaxter 2007).

Genomic analyses of *Wolbachia* have revealed the presence of biosynthetic pathways absent in their nematode hosts. These include the synthesis of heme, flavin adenine dinucleotide (FAD), riboflavin (vitamin B2), glutathione, purines, pyrimidines, and other nucleotides (Bain et al. 2008; Bouchery et al. 2013). These metabolic capabilities enable *Wolbachia* to fulfill the nutritional requirements of essential processes such as embryogenesis, oogenesis, and overall development (Foster et al. 2005). In return, nematode hosts supply *Wolbachia* with coenzyme A, nicotinamide adenine dinucleotide (NAD), biotin, ubiquinone, folate, lipoic acid, and pyridoxal phosphate (compounds vital for bacterial growth and maintenance) (Foster et al. 2005). The absence of *Wolbachia* has been shown to induce apoptosis in cells of embryos, microfilariae, and L4‐stage juveniles, likely due to a deficiency in key metabolites that otherwise suppress programmed cell death (Landmann et al. 2011). Beyond its mutualistic role, *Wolbachia* contributes to the pathology of filariasis in humans. Upon nematode death (either through pharmacological treatment or natural causes), *Wolbachia* is released, triggering inflammatory responses and exacerbating immunopathology (Mukherjee et al. 2017; Taylor et al. 2005). Beyond filarial nematodes, *Wolbachia* has also been identified in three plant‐parasitic nematode species (*Pratylenchus penetrans*, *Radopholus arabocoffeae*, and *Radopholus similis* ), as well as in the insect–parasitic nematode *Howardula* (Brown et al. 2016; Dudzic et al. 2022; Haegeman et al. 2009). Phylogenomic analyses suggest that these strains form an early‐diverging lineage within the *Wolbachia* clade, implying an ancient association with nematodes, particularly among plant‐parasitic taxa (Brown et al. 2016; Dudzic et al. 2022). The genome of the *Wolbachia* strain from *P. penetrans* displays notable distinctions from those found in filarial nematodes (Brown et al. 2016). For instance, it retains only one of six riboflavin biosynthesis genes and lacks the necessary genes for biotin and thiamine biosynthesis (Brown et al. 2016). In contrast, the *Wolbachia* genome from *Howardula* is highly reduced (approximately 35% smaller than the smallest known *Wolbachia* genomes in nematodes) and contains no evidence of recent horizontal gene acquisitions (Dudzic et al. 2022). However, it preserves a complete heme biosynthetic pathway, suggesting that this function may be central to its persistence and evolutionary stability in nematode hosts.

### 2.4 Entomopathogenic Nematodes

Among the various reported mutualisms, entomopathogenic nematodes (EPNs) stand out for their symbiosis with entomopathogenic bacteria (Dillman et al. 2012; Gaugler 2002; Stock and Blair 2008). Entomopathogeny has evolved multiple times within the order Rhabditida, with *Steinernema* and *Heterorhabditis* being key genera that maintain specific associations with the bacteria *Xenorhabdus* and *Photorhabdus*, respectively (Poinar and Grewal 2012; Trejo‐Meléndez et al. 2024). These symbiotic bacteria are central to the EPN life cycle, acting as “biological weapons” to subdue insect hosts (Adams et al. 2006). The life cycle of EPNs begins with the infective dauer larvae, a stress‐resistant resting stage that carries internally their bacterial symbionts. These larvae actively search for insect hosts in the soil; once located, they penetrate the insect's hemocoel and then release their symbionts, either via regurgitation or defecation. The bacteria rapidly proliferate within the host, causing septicemia that leads to the insect's death. The nematodes then feed on the bacteria and decomposing host tissue, allowing them to develop, reproduce, and ultimately disperse to new hosts (Ciche et al. 2006). This tightly integrated interaction illustrates a stable evolutionary and ecological mutualism (Ciche et al. 2006; Stock and Blair 2008).

The absence of these bacterial symbionts significantly impairs EPN fitness. Axenic (bacteria‐free) nematodes exhibit reduced growth and reproductive success compared to symbiotic individuals (McMullen et al. 2017; Park et al. 2011). Multiple genomic studies have confirmed the mutualistic nature of this relationship (Ciche et al. 2006; Ogier et al. 2020; Waterfield et al. 2009). For example, bacterial genomes encode traits essential for symbiosis, including adhesion to the nematode gut and biosynthesis of key nutrients (Brehélin et al. 1993; Goodrich‐Blair 2007). Additionally, bacteria play a key role in overcoming insect immune defenses. Synthesis of toxins and modulating immune factors suppress host immune response, promoting insect death (Castillo et al. 2011; Eleftherianos et al. 2018). Before releasing their mutualistic bacteria, nematodes employ immune evasion strategies to avoid detection by the insect host. One such strategy involves cloaking themselves with host‐derived antigens bound to their cuticle, effectively disguising their presence (Brivio and Mastore 2018). Subsequently, symbiotic bacteria such as *Xenorhabdus* are released into the insect hemocoel, where they secrete toxins that suppress the host immune response (Goodrich‐Blair and Clarke 2007). As the bacteria support insect infection and killing, the nematode contributes by producing specific amino acids needed by the bacteria or by evolving specialized structures for bacterial storage and transmission (Goodrich‐Blair 2007). Together, these findings posit the EPN‐bacteria relationship as a valuable model for studying the evolution of mutualism and parasitism (Trejo‐Meléndez et al. 2024). However, despite the existence of over 100 *Steinernema* and 21 *Heterorhabditis* species (Bhat et al. 2020), fewer than five species have been extensively studied in this context. Moreover, most studies have focused narrowly on the core symbionts, often neglecting the broader microbial community (Ogier et al. 2020).

**FIGURE 2:**
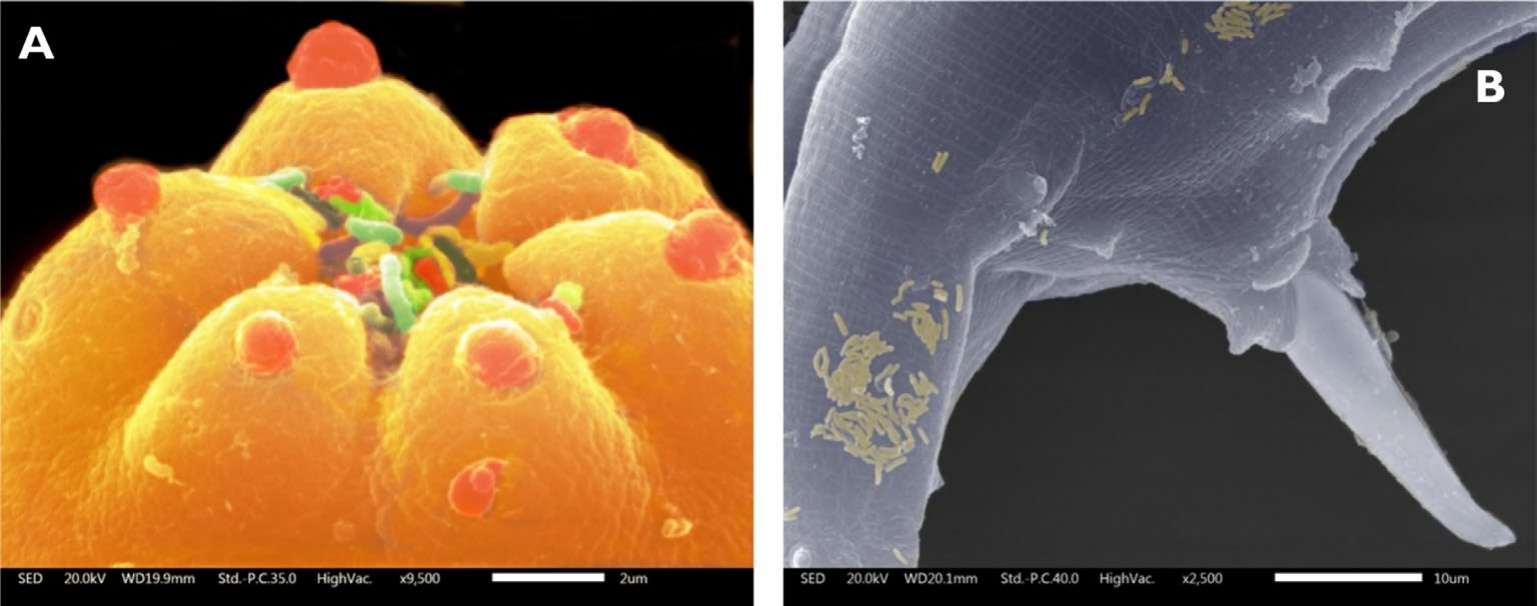
(A) *Rhabditis regina* with bacterial cells visible on the buccal apparatus, captured during feeding. (B) Bacterial cells attached to the nematode's body surface. The prominent conical structure in panel (B) is the male spicule, used for fertilizing females. Images courtesy of Orlando Hernández (Microscopy Laboratory, ENES Morelia, UNAM) and Jorge Contreras‐Garduño.

Recent studies have shown that infective dauer larvae of several *Steinernema* species harbor a diverse microbiota, resembling that found in other entomopathogenic rhabditids (Jiménez‐Cortés et al. 2016; Park et al. 2011). For instance, Ogier et al. (2020) reported that *Xenorhabdus* spp., the core symbiont, coexists with a wide array of Proteobacteria, including *Pseudomonas*, *Stenotrophomonas*, *Alcaligenes*, *Achromobacter*, *Pseudochrobactrum*, *Ochrobactrum*, *Brevundimonas*, and *Deftia*. Notably, *Pseudomonas protegens* and *P. chlororaphis* exhibited independent entomopathogenic activity, suggesting that virulence in EPNs may arise from additive or synergistic interactions among multiple bacterial taxa (Ogier et al. 2020). Similarly, in *Rhabditis regina*, both infective dauer and L2–L3 larvae were found to carry a diverse bacterial community, including *Clostridium* (Jiménez‐Cortés et al. 2016). These bacteria were acquired under laboratory conditions, likely originating from contaminated meat, highlighting the nematodes capacity to incorporate and establish novel environmental bacteria into their microbiome (Jiménez‐Cortés et al. 2016; Figure 2). Such findings underscore the pivotal role of microbiota in the ecology and evolution of insect parasitism and highlight the need for further research into the contribution of nematodes to their own ecological plasticity (Jarriault and Gally 2024), as well as the role of their associated bacterial communities in shaping this plasticity.

### 2.5 Pasteuria and Its Antagonist Role with Nematoda

The symbiotic association of nematodes and bacteria encompass both mutualistic and antagonistic interactions. Among the latter, the Gram‐positive, filamentous, endospore‐forming bacterium *Pasteuria* spp. is a well‐documented endoparasite of nematodes (Ciancio 1995; Davies et al. 2023). Although *Pasteuria* has been isolated from over 300 nematode species (including predatory, free‐living, fungivorous, and bacterivorous taxa), its parasitism is most extensively studied in plant‐parasitic nematodes (Chen and Dickson 1998; Davies et al. 2023; Sturhan et al. 2005). In soil, *Pasteuria* initiates its life cycle from dormant endospores through a three‐stage process: (1) attachment and germination, (2) rhizoid development and exponential proliferation, and (3) sporogenesis (Davies et al. 1991, 2011). Endospore attachment is highly host‐specific; strains typically infect only specific nematode populations, often failing to parasitize even conspecific individuals from different geographic origins (Davies et al. 1988; Stirling 1985). This specificity is mediated by intricate interactions between endospore surface epitopes and nematode cuticle receptors (Davies et al. 2023; Spiegel et al. 1996). Evidence suggests that *Pasteuria* parasitism may have influenced the evolution of reproductive strategies in plant‐parasitic nematodes, particularly the divergence between amphimictic and parthenogenetic modes (Davies et al. 2023). In amphimictic species such as *Heterodera*, *Pasteuria* germinates early and produces relatively few mature endospores (~10^3^), whereas in parthenogenetic species such as *Meloidogyne* spp., germination is delayed, allowing for the formation of significantly more endospores (~10^6^–10^7^; Davies et al. 2023). According to models of the evolution of sexual reproduction (Hamilton 1980), genetic recombination may buffer parasitism effects, though its efficacy depends on the genomic loci involved and frequency‐dependent interactions. Genomic analyses of *Meloidogyne* spp. indicate a hybrid origin involving divergent parental genomes, with species divergence facilitated by non‐crossover recombination events (Szitenberg et al. 2017). These dynamics suggest that *Pasteuria* parasitic strategies may exploit patterns of fluctuating linkage disequilibrium, which are more prevalent in parthenogenetic than in sexually reproducing nematode lineages (Davies et al. 2023). Overall, the *Pasteuria*‐nematode interaction offers a valuable model for studying host–parasite co‐evolution, including hypotheses such as the Red Queen. Adaptations such as diverse adhesins that facilitate cuticle attachment likely represent *Pasteuria* evolutionary responses to host defenses. Investigating how nematodes counter these strategies (including the potential role of their associated microbiota) is a promising avenue for future research. Notably, certain bacterial taxa in the nematode's external microbiome may provide protective functions against fungal and bacterial infections in soil environments (Topalović and Vestergård 2021).

Microbial partnerships have likely facilitated the diversification of nematode lineages by enabling adaptations to both parasitic and free‐living lifestyles. Whether mutualistic or antagonistic, such associations reflect deep co‐evolutionary dynamics, with nematode survival and ecological success often depending on microbial partners. Long‐term symbioses are made possible by the co‐inherited genetic architecture of both partners and the persistence of selective pressures favoring such interactions. Compared to other nematodes, the microbiota of *C. elegans* has received disproportionate attention (Gerbaba et al. 2017; Johnke et al. 2020; Shapira 2017; Zhang et al. 2017). Numerous studies have investigated the genomic determinants of microbiota assembly and function in *C. elegans* (Berg et al. 2019; Haçariz et al. 2021; Ortiz et al. 2021; Taylor and Vega 2021; Zhang et al. 2021; Zimmermann et al. 2024), contributing significantly to the broader theoretical framework of host–microbiota interactions. As such, *C. elegans* remains a key model for understanding the influence of microbiota on host fitness across generations and environmental contexts.

## 3 *C. elegans* Microbiota

The free‐living nematode *C. elegans* completes its life cycle in saprophytic environments such as rotting fruit and decaying plant matter. As a bacterivore, it feeds on the bacterial communities that thrive in these habitats. Recent studies have provided important insights into the composition, maintenance, and functional roles of its microbiota (Dirksen et al. 2016; Gerbaba et al. 2017). Due to its relatively low microbiota diversity, *C. elegans* serves as a tractable model for experimental manipulation of bacterial communities. This has enabled the elucidation of many mechanisms underlying microbiota assembly and host–microbe interactions. Furthermore, since bacteria constitute the nematode's primary food source, the direct effects of individual bacterial strains or consortia on host physiology and fitness can be readily tested (Frézal and Félix 2015). Although *Caenorhabditis* species are predominantly saprophytic, alternative lifestyles have been documented. For instance, *Caenorhabditis briggsae* has been reported to exhibit entomopathogenic behavior, a trait conferred by its association with entomopathogenic bacteria (Abebe et al. 2011; Abebe‐Akele et al. 2015). These findings deserve a deep exploration but highlight the influence of ecological context on microbiota structure and function, shaped by factors such as biotic interactions, nutrient availability, and habitat characteristics (Schulenburg and Félix 2017). Understanding the ecological niche of nematodes is thus critical for interpreting how microbiota contributes to host ecology and evolutionary dynamics. Three recent studies have characterized the core microbiota of *C. elegans* (Table 1) (Berg, Zhou, and Shapira 2016; Dirksen et al. 2016; Samuel et al. 2016). A meta‐analysis identified a consistent core of twelve bacterial families, including Gammaproteobacteria (Enterobacteriaceae, Pseudomonadaceae, Xanthomonadaceae) and Bacteroidetes (Sphingobacteriaceae, Weeksellaceae, Flavobacteriaceae; Zhang et al. 2017). Common genera include *Pseudomonas*, *Stenotrophomonas*, *Ochrobactrum*, and *Sphingomonas*. Despite being collected from diverse geographic locations and substrates, nematode populations share a remarkably similar microbiota composition. Interestingly, bacteria with apparently beneficial effects on host fitness often occur at relatively low abundances. For example, although Acetobacteriaceae is correlated with increased *C. elegans* population growth, it is less abundant than many other taxa (Berg, Zhou, and Shapira 2016). Similar patterns are observed for other potentially beneficial groups, including Proteobacteria (e.g., Moraxellaceae, Comamonadaceae and Rhodobacteraceae) and Actinobacteria (e.g., Microbacteriaceae, Actinomycetales).

**TABLE 1:** Core microbiota and its effect on *C. elegans* life‐history traits.

Source of *C. elegans* nematodes	Collected in	Main bacteria taxa	Bacterial effects	References
Wild worms collected from France/Spain locations	Apples, compost, inside invertebrates, plant stems	Proteobacteria (Enterobacteriacea, Pseudomonadacea, Xanthomonadacea, Brucellaceae, Sphingomonadaceae)	Population increases correlated to α‐Proteobacteria presence Ubiquity of *Bacteroidetes* and other pathogens produce non‐prolific dauer larvae	Samuel et al. (2016)
Wild worms collected from north Germany	Apples, oranges, prickly pear, snails, black walnut stems	Proteobacteria (Enterobacteriaceae, Acetobacteriaceae), Bacteroidetes, Firmicutes, Actinobacteria	Population increases under normal and stressful conditions. Defense against pathogenic fungi	Dirksen et al. (2016)
N2 Laboratory strain	Compost soils produced by different elements	Proteobacteria (Enterobacteriaceae, Pseudomonadaceae, Xanthomonadaceae, Burkholderiaceae, Aeromonadaceae, Alcaligenaceae, Rhizobiaceae), Bacteroidetes, Firmicutes	Protection against pathogens that may changes with lifespan. This feature is species‐specific	Berg, Stenuit, et al. (2016); Berg, Zhou, and Shapira (2016)

The studies discussed above provide a foundation for investigating the diversity and evolutionary dynamics of nematode‐associated microbiotas. However, broader taxonomic sampling is needed to fully understand microbiota variation across nematode species. To date, only the study by Dirksen et al. (2016) has compared the microbiotas of *C. elegans* , *Caenorhabditis remanei*, and *C. briggsae* . They reported that *C. elegans* harbored a microbiota distinct from *C. remanei* but not significantly different from *C. briggsae* (a result that may be influenced by the limited sample size for the latter; Dirksen et al. 2016). These findings suggest the existence of species‐specific microbiotas, with similarities emerging primarily at higher taxonomic levels, such as the family level. Nonetheless, additional studies encompassing a wider array of nematode species are essential to confirm these patterns. Given that many nematodes inhabit similar environments (e.g., decomposing plant material), some overlap in microbiota composition might be expected. However, lifestyle differences, such as saprophytic, predatory, or parasitic modes of life, impose distinct physiological demands on hosts, which may in turn be met or mediated by their associated microbiota (Maher et al. 2017; Shapira 2017). It is therefore plausible that beneficial bacteria are selectively maintained for functions that enhance host performance, such as resource exploitation, detoxification, or defense against pathogens and environmental stressors. At the same time, microbiota composition is shaped by host‐imposed selective pressures, including feeding behavior, immune responses, gut niche availability, and bacterial competition via antimicrobial production (Berg, Stenuit, et al. 2016; Sze et al. 2020). Understanding these co‐adaptive processes between nematodes and their microbiota is key to elucidating the mechanisms of community assembly (Singh and Luallen 2024; Zilber‐Rosenberg and Rosenberg 2008).

Experimental evolution offers a promising approach for testing hypotheses about microbiota dynamics, allowing researchers to explore factors that influence microbiota structure and function both within and across host generations (Gerbaba et al. 2017). Another important, yet often overlooked, aspect is microbiota variation across the nematode life cycle. Most studies to date have analyzed mixed‐stage populations (e.g., larvae and adults in Berg, Stenuit, et al. 2016 and Samuel et al. 2016) or have focused on limited stages such as L4 larvae and adults (e.g., Dirksen et al. 2016). A life stage–specific approach to microbiota characterization could provide critical insights into how microbial communities respond to shifting physiological, immunological, and reproductive demands throughout development. For instance, dauer larvae, characterized by distinct anatomical and metabolic traits, may exert unique selective pressures that favor specific microbial taxa (Cassada and Russell 1975; Diaz et al. 2015; Dolan et al. 2002). Applying a life‐history perspective would not only improve our understanding of how microbiota composition and function shift over time but also illuminate the biological roles of community attributes such as species richness and evenness and their impact on nematode fitness and ecology (Shapira 2017).

## 4 Function of the *C. elegans* Microbiota

Microbiota‐derived metabolites play essential roles in supporting *C. elegans* across multiple functional domains, including biochemical (e.g., complementing metabolic pathways, detoxification), physiological and behavioral (e.g., maintaining homeostasis), and immunological processes (e.g., pathogen defense; Akduman et al. 2020; Montalvo‐Katz et al. 2013; Sommer et al. 2017; Urquiza‐Zurich et al. 2023; Zimmermann et al. 2020). In natural environments, *C. elegans* alternates between aerobic and anaerobic conditions, and specific metabolic demands are met through interactions with its microbiota (Zimmermann et al. 2020). Microbial synthesis of essential nutrients has been shown to directly influence nematode growth and reproduction (Berg, Zhou, and Shapira 2016; Block and Shapira 2015). For instance, in *Pristionchus pacificus*, microbiota‐derived metabolites modulate neuroendocrine signals (such as TGF‐β ligands) that are critical for development and fertility (MacNeil 2022; Rae et al. 2008). Similarly, nematode behavior is shaped by microbial metabolites that mimic neurotransmitters (Samuel et al. 2016; Shtonda and Avery 2006; Weisskopf et al. 2019). Since behaviors like foraging, mating, and pathogen or predator avoidance are key life‐history traits, the microbiota's influence on behavioral plasticity has important ecological and evolutionary implications (Gordon 2011; Urquiza‐Zurich et al. 2023). Exploring these microbial contributions offers a promising avenue for understanding the costs and benefits of harboring specific microbiotas under different ecological contexts. Microbiota‐mediated enhancement of *C. elegans* immune responses is a well‐established area of research (Berg, Zhou, and Shapira 2016; Montalvo‐Katz et al. 2013). Protective bacterial taxa have been identified at both the species and strain level. For example, some strains of *Pseudomonas* confer resistance against the nematicidal fungus *Drechmeria coniospora*, suggesting strain‐specific recognition and immune priming mechanisms (Dirksen et al. 2016). Dirksen et al. (2016) also isolated *Pseudomonas* strains that inhibited the growth of six fungal pathogens, including *D. coniospora* (Lebrigand et al. 2016). Similarly, *Enterobacter cloacae* strains isolated from *C. elegans* and *C. briggsae* provide protection against *Enterococcus faecalis* , but this protection is species‐specific: a strain protective in *C. elegans* does not confer the same benefit in *C. briggsae* , and vice versa (Berg, Zhou, and Shapira 2016). Understanding the mechanisms underlying such species‐specific immune interactions and how they arise are key areas for future research (Kissoyan et al. 2019; Radeke and Herman 2021).

It has been hypothesized that certain bacteria, such as members of the *Pseudomonadales*, may be coevolving with *C. elegans* (Berg, Zhou, and Shapira 2016). Testing this hypothesis through comparative and phylogenetic approaches could provide insights into long‐term host–microbe associations. One of the most intriguing challenges in host–microbiota research is understanding how *C. elegans* balances coexistence between mutualistic and pathogenic microbes. Investigating the mechanisms that regulate this balance is crucial for identifying how hosts retain or eliminate specific bacterial taxa. By unraveling these dynamics, we can better understand how host phenotypes and genotypes shape, and are shaped by, microbial community structure. As highlighted in this review, one particularly significant pathway influenced by bacterial metabolites is insulin signaling, due to its known role in regulating key fitness components such as growth, development, stress resistance, and lifespan (Flatt and Heyland 2011; Zera and Harshman 2001). Microbiota effects on host evolution can thus be inferred through their impact on life‐history traits. These traits (such as growth rate, survival, and reproductive output) provide a framework to assess individual performance and adaptation across environmental conditions. Indeed, multiple studies have demonstrated associations between microbiota composition and *C. elegans* phenotype, including developmental rate and fecundity (Taylor and Vega 2021; Zhang et al. 2021). Disruption of beneficial host–microbe associations can also have detrimental effects. For instance, when the core symbiont *Xenorhabdus* is removed from *Steinernema* spp., the nematode's reproductive capacity and virulence are significantly diminished (McMullen et al. 2017). Such findings illustrate how selection may act on host phenotypes that promote the maintenance and transmission of beneficial microbial communities across generations.

## 5 Microbiota and *C. elegans* Life History

Numerous studies have examined the effects of individual bacterial strains or simple consortia on *C. elegans* life‐history traits. However, the extent to which the broader microbiota shapes nematode phenotype remains unclear. Samuel et al. (2016) demonstrated that *C. elegans* population growth is influenced by the composition of the microbial community in its substrate. Microhabitats enriched in α‐Proteobacteria (particularly members of the Enterobacteriaceae and Acetobacteriaceae families) supported higher nematode proliferation compared to those dominated by Bacteroidetes (e.g., Flavobacteriaceae) or γ‐Proteobacteria (e.g., Xanthomonadaceae, Pseudomonadaceae). These findings suggest that microbiota effects are context‐dependent, shaped by both the nematode's microhabitat and complex microbial community interactions. Dirksen et al. (2016) experimentally tested the effects of multi‐species bacterial consortia on *C. elegans* physiology, identifying several beneficial Proteobacteria, including *Gluconobacter*, *Enterobacter*, *Pseudomonas*, *Acinetobacter*, *Ochrobactrum*, *Providencia*, and *Lactococcus*. Conversely, they also identified detrimental taxa such as the Bacteroidetes *Chryseobacterium* and *Sphingobacterium* and potentially pathogenic γ‐Proteobacteria such as *Xanthomonas* and *Stenotrophomonas*. Despite these advances, many studies remain focused on isolated life‐history traits (Akbar et al. 2020; Diaz et al. 2015; Gould et al. 2018; Hoang et al. 2019; Kolodny and Schulenburg 2020; Macke et al. 2017; Santos et al. 2023), often without comparative analysis across environmental contexts or populations. As a result, our understanding of how microbiota influences variation in life‐history strategies across natural populations remains limited. The most commonly studied traits include growth rate (Akbar et al. 2020; Gould et al. 2018; Hoang et al. 2019; Kolodny et al. 2020; Macke et al. 2017), survival and longevity (Donato et al. 2017; Gusarov et al. 2013; Ikeda et al. 2007; Kim 2013; Kissoyan et al. 2022; Shin et al. 2020; Troemel et al. 2006; Virk et al. 2012, 2016), and reproductive output (Hoang et al. 2019; Kissoyan et al. 2022; Lee et al. 2020a, 2020b; Stuhr and Curran 2020; Yang et al. 2019). Some research has begun to explore the molecular mechanisms underlying these trait changes, with particular emphasis on the insulin‐like signaling pathway. This pathway plays a central role in regulating key biological processes (including metabolism, immunity, and development) that directly affect life‐history traits. In the following section, we explore these mechanistic insights and evaluate their implications in the context of life‐history theory (Table 2).

**TABLE 2:** List of bacteria that influence *C. elegans* nematode life‐history traits.

	Bacteria	Function	References
Survival/longevity	*Bacillus subtilis*	Favors longevity by biofilm generation and nitric oxide (NO) production	Gusarov et al. (2013) Donato et al. (2017) Hoang et al. (2019)
	*Pseudomonas mendocrina*	Increase pathogen resistance by induction of immune genes regulated by the p38 MAPK pathway	Montalvo‐Katz et al. (2013)
	*Lactobacillus rhamnosus*	Display antibiotic and antioxidant activity that counter negative effects of pathogens	Azat et al. (2016)
	*Enterococcus faecalis*	Protect against *Staphylococcus aureus* by antimicrobial superoxide synthesis	King et al. (2016)
	*L. acidophilus*	Provide protection against pathogen response and stress‐response mediated by p38 MAPK.	Kim and Mylonakis (2012) Li et al. (2018)
	*Lactobacillus y Bifidobacteria*	Increase longevity by p38 MAPK pathway, which stimulates defense mechanisms that protect against pathogens	Ikeda et al. (2007) Komura et al. (2013)
	*Bifidobacteria* spp.	Activate *skn*‐1 gene involved in homeostatic and aging pathways	Komura et al. (2013)
Growth	*Xanthomonas* sp.	Induction of expression of 12 genes linked to cuticle shedding	Sthur and Curran (2021)
	*Methylobacterium* sp.		
	*Sphingomonas* sp.		
	*Pseudomonas* spp.	Vitamins synthesis	Yang et al. (2019) Dirksen et al. (2020); Zimmermann et al. (2020)
	*Ochrobactrum* spp.		
	*L. acidophilus*	Provide protection mediated by β‐catenin pathways. This pathway favors embryonic development, including cell migration and polarity.	Kim and Ausubel (2005); Kim and Mylonakis (2012); Zhi et al. (2017)
	*Acinetobacter* spp.	Degrade trans‐3‐hidroxiproline to hidroxiproline, which is part of collagen IV, a main component of pharynx, intestine and cuticle extracellular matrix	Zimmermann et al. (2020)
	*Rhodococcus* spp.		
Reproduction	*Achromobacter* spp.	Enhances 2.5 fold larvae production	Dirksen et al. (2016)
	*Comamonas* spp.		
	*Pseudomonas* spp.		
	*Pseudomonas lurida*	Enhances larvae production by 22% y 31%, respectively	Kissoyan et al. (2022)
	*Pseudomonas fluorescens*		

### 5.1 Longevity

Longevity is one of the most extensively studied traits in *C. elegans* , largely due to its relevance as a model for understanding aging in humans (Kim 2013; Tissenbaum 2015). Numerous bacterial species have been shown to influence lifespan and aging processes in *C. elegans* (Gusarov et al. 2013; Khan et al. 2018; Samuel et al. 2016; Table 2). Within a life‐history framework, however, longevity is intimately tied to other attributes like juvenile and adult mortality rates, which in turn define early or late maturing strategies (Stearns 1992). Low mortality rates result in extended lifespan, attaining greater individual size and more reproductive events (Poulin 2007; Stearns 1992). Although this suggests that microbiota contributions are context‐dependent on environmental pressures, microbiota would enable optimal resource allocation, utilization, and countering environmental threats, thus supporting nematode fitness. Consequently, given the close link between longevity and survival, research often simultaneously examines the role of microbiota in enhancing host resistance to stress and pathogens. In *C. elegans* , *Bacillus subtilis* is particularly notable for promoting longevity under stressful conditions, such as thermal shock (Donato et al. 2017; Gusarov et al. 2013; Hoang et al. 2019). This effect is mediated by bacterial metabolites that modulate the conserved insulin signaling pathway involving DAF‐2, DAF‐16, and HSF‐1 (Donato et al. 2017). Moreover, over 20 strains of lactic acid bacteria (probiotics) have been shown to extend *C. elegans* lifespan by enhancing defense mechanisms against pathogenic microbes (Khan et al. 2018; Table 2). These protective effects suggest that natural selection may favor behaviors or recognition systems (such as the detection of specific volatile metabolites) that facilitate the acquisition of beneficial microbiota. While most evidence comes from laboratory studies, these findings provide a valuable framework for understanding the ecological and evolutionary dynamics of host–microbe interactions.

### 5.2 Growth

Body size is a key trait influencing a wide array of biological and behavioral characteristics (Stearns 1992). In nematodes, body size correlates strongly with reproductive output and population growth potential (Morand et al. 1996; Skorping et al. 1991). Larger individuals (both male and female) tend to have greater mating success and enhanced competitive abilities, which may confer advantages in resource acquisition and predator avoidance (Canales‐Lazcano et al. 2019). Developmental rate is often linked to body size and can be shaped by habitat conditions. In *C. elegans* , life in ephemeral, resource‐limited (saprophytic) environments favors rapid development to ensure reproduction before resource depletion. However, life‐history theory predicts that this accelerated growth often comes at a cost: smaller adult size and reduced reproductive capacity (Stearns 1992). By contrast, parasitic nematodes in vertebrate hosts can afford slower development, achieving larger body sizes due to abundant internal resources (Harvey and Keyme 1991; Skorping et al. 1991). Yet, host immune responses may drive earlier development and reproduction as an adaptive strategy (Sorci et al. 2003). These patterns suggest that body size is a trait under strong selection (Babayan et al. 2010) and highlight the potential role of the microbiota in mediating adaptive growth responses.

Optimal growth is generally associated with nutritional quality (Stuhr and Curran 2020). In *C. elegans* , bacteria serve as both a food source and potential symbionts, making it critical to distinguish between those that are digested and those that colonize the gut (Diaz et al. 2015; Kim 2013). This distinction helps determine whether observed physiological effects result from nutritional input or from symbiotic interactions. Despite its importance, relatively few studies have evaluated the nutritional value of bacteria, particularly those occurring in the natural habitats of *C. elegans* . Stuhr and Curran (2020) compared bacterial genera such as *Xanthomonas*, *Methylobacterium*, and *Sphingomonas*, which differ markedly in their content of glucose, glycerol, glycogen, water, and triglycerides. Transcriptomic analyses of nematodes fed on these bacteria revealed distinct “transcriptional signatures” yet also shared expression of 12 genes involved in cuticle molting (e.g., *sqt‐2*, *sqt‐3*, *ptr‐4*, *mlt‐7*, *rol‐6*, *noah‐1*; Zečić et al. 2019). These differences were reflected in nematode body size, which varied significantly among treatments, consistent with other findings (Nagashima et al. 2017; Tuck 2014). Notably, nematodes exhibited increased growth relative to those fed with *Escherichia coli* (Stuhr and Curran 2020). However, the bacteria in this study were fully digested, indicating that the observed growth enhancement was due to their nutritional composition. In contrast, when *C. elegans* is colonized by a bacterial consortium, growth responses are often driven by symbiotic interactions. These include upregulation of genes involved in metabolism, dietary responses, and the insulin signaling pathway (e.g., *daf‐2*, *daf‐16*), as well as GATA and E‐box transcription factors (Dirksen et al. 2016; Yang et al. 2019; Zhang et al. 2017). The former are known to regulate immunity, intestinal development, and aging (Shapira et al. 2006), while the latter influence immunity and muscle development (Grove et al. 2009). Additionally, some bacteria provide essential amino acids and vitamins that directly support nematode growth and fertility (Dirksen et al. 2016; Yang et al. 2019; Zimmermann et al. 2020; Table 2). Understanding how specific microbial communities influence body size and development may offer new insights into host–microbe co‐evolution and life‐history trade‐offs in nematodes.

### 5.3 Reproduction

Within the framework of life‐history theory, reproduction is costly and encompasses both the quantity and quality of offspring (Roff 2002; Stearns 1992; Victoria Herreras et al. 2007). The role of the microbiota in *C. elegans* reproduction has been only superficially explored. Most studies focus on changes in total progeny production, reporting either increases or decreases in offspring number (Dirksen et al. 2016; Stuhr and Curran 2020; Table 2). For instance, *Methylobacterium* and *Sphingomonas* have been shown to downregulate genes associated with reproductive functions (Stuhr and Curran 2020). While the underlying mechanisms remain unclear, it is likely that bacterial metabolites influence reproductive output through modulation of key signaling pathways, such as the insulin signaling cascade (Zimmermann et al. 2020). The significance of microbiota in nematode reproduction becomes more apparent when considering heritability. Vertically transmitted microbes can substantially influence offspring fitness (Freitak et al. 2014). In insects, for example, vertical transmission enables transgenerational immune priming, where parental exposure to pathogens enhances offspring resistance (Sheehan et al. 2020). A similar mechanism may exist in nematodes, and identifying bacterial strains that promote transgenerational resistance presents an exciting avenue for future research (Freitak et al. 2014). All this evidence suggests that microbiota would influence nematode reproduction by synthesizing essential nutrients (that improve offspring quality and quantity) or complementing signaling pathways linked to reproduction (as physiological and behavioral signals during mating). Exploring this issue is crucial and would expand the understanding of the role of microbiota on nematode reproduction. Also, within life‐history theory, reproduction decreases through lifespan, so it would be interesting to track microbiota adjustments to explain early and later reproduction, especially for iteroparous organisms like many nematodes. In addition, the dual aspect of offspring quality and quantity is rarely addressed in microbiota studies, leading to an incomplete understanding of microbial effects on reproductive fitness. Early‐maturing individuals may produce large numbers of offspring, but potentially at the cost of reduced offspring quality or viability. Life‐history theory predicts trade‐offs among fitness traits, and reproduction is no exception. Therefore, in the following section, we examine how the microbiota may influence these life‐history trade‐offs, shedding light on its broader evolutionary implications.

## 6 Microbiota Modifies Nematode Life History Trade‐Offs

Life‐history traits require resource investment for their development and maintenance. However, resource availability is inherently limited, necessitating optimal allocation among competing traits (Stearns 1989; Zera and Harshman 2001). Symbiotic associations can further complicate this balance by imposing additional metabolic costs, as hosts must divert resources from other physiological functions to maintain these interactions (Hoang et al. 2021; Macke et al. 2017). As a result, microbial symbionts do not always confer net benefits to their hosts (Jacob et al. 2015). In *C. elegans* , life‐history trade‐offs are modulated by microbial symbionts, although the underlying mechanisms remain poorly understood. For instance, colonization by *Methylobacterium* and *Sphingomonas* improved development and extended lifespan but reduced reproductive output by 25% and 10%, respectively. In contrast, *Xanthomonas* promoted faster development without affecting reproduction, yet decreased longevity (Stuhr and Curran 2020). Similarly, *Pseudomonas* species enhanced fecundity at the cost of reduced lifespan (Kissoyan et al. 2022). Further evidence of microbiota‐mediated trade‐offs comes from studies using synthetic microbial communities designed to mimic those found in wild *C. elegans* . These experiments revealed increased developmental rates but significantly reduced fecundity and thermal stress resistance (Slowinski et al. 2020). These findings suggest that specific microbiota compositions (shaped by environmental microcosms) can drive distinct alterations in nematode life‐history traits. One of the most studied life‐history trade‐offs involves the balance between offspring quantity and quality (Stearns 1989, 1992; Zera and Harshman 2001). However, in nematodes, this specific trade‐off remains largely unexplored.

## 7 Microbiota as a Driver of Nematode Adaptation

By influencing life‐history traits, microbiota can shape the adaptive capacity of their hosts (Duperron et al. 2013; Thrall et al. 2007). Under adverse or novel environmental conditions, specific host–microbiota associations may emerge that enhance the fitness of both partners (Davitt et al. 2011). These interactions can facilitate host expansion into previously inaccessible environments (Douglas 2014). When such selective pressures persist over evolutionary timescales, co‐adaptation between hosts and their microbiota is likely (Hoang et al. 2019, 2021; Kitano and Oda 2006; Klepzig et al. 2009). For instance, *B. subtilis* enhanced *C. elegans* reproductive output after 20 generations of exposure to heat shock treatments (Hoang et al. 2019). The authors suggest this increased thermal tolerance arose from the evolution of stress‐response genes within the host (Hoang et al. 2021). Notably, benefits extended to the bacteria as well, which showed increased abundance. In another study, *C. elegans* ‐associated microbiota improved the nematode's tolerance to cadmium, along with enhanced larval production and lifespan (Lee et al. 2020b). Although explicit trade‐offs were not observed, it is essential to examine additional life history traits (such as offspring quality) to better understand the fitness consequences of these associations. The study also found upregulation of genes linked to chemical stress and immune responses, indicating that gene expression changes underlie the observed increases in resistance (Lee et al. 2020b). These findings underscore the role of complex gene networks in regulating nematode life‐history under stress. Investigating such networks during environmental challenges could elucidate how phenotypes are shaped through host–microbiota interactions. EPNs provide an excellent model for studying how microbiota influences host adaptation. EPNs form highly specific symbioses in which many essential functions depend on a core bacterial partner. Remarkably, a study on *Heterorhabditis downesi* demonstrated that replacing its native symbiont *Photorhabdus* improved desiccation resistance and increased larval production (Maher et al. 2017). This is an unusual case, as symbiont replacement in EPNs typically has negative fitness consequences. These results suggest that core symbionts may be functionally replaceable under certain conditions, although the long‐term stability and resilience of such modified symbioses require further investigation. Despite its significance, the role of gut microbiota in host adaptation remains an underexplored area. However, emerging evidence indicates it holds considerable potential. Studying adaptation through the lens of host–microbiota interaction offers a promising avenue for understanding metazoan evolution. Nematodes are ideal models due to their ecological diversity and ability to transition between free‐living and parasitic lifestyles. Moreover, nematodes are among the few metazoans in which HGT has been clearly demonstrated (Danchin and Rosso 2012; Dieterich and Sommer 2009; Mayer et al. 2011). This phenomenon represents a major shift in our understanding of evolutionary processes, highlighting the importance of non‐vertical inheritance in shaping life‐history evolution.

## 8 Fungi, Viruses and Protozoa, the Dismissed Partners

In soil, fungi and nematodes interact in mutualistic and antagonistic relationships, with the latter being the most studied (Zhang et al. 2020). Many studies have addressed pathogenic fungi biology from their nematode hosts (Hsueh et al. 2013; Lebrigand et al. 2016; Lee et al. 2020a; Nordbring‐Hertz 1979). As a biological control candidate, fungi associated with nematodes have been studied mainly in plant‐parasitic nematodes from *Heterodera* and *Globodera* species (Hu et al. 2020; Jumbam et al. 2024). There is evidence that recognizes fungi as a main factor regulating population densities of cyst nematodes (Liu and Chen 2003). Importantly, cysts are known to harbor soil‐inhabiting fungi, acting as an important reservoir for plant pathogenic fungi (Mclean and Lawrence 1995). Besides application to control plant‐parasitic nematodes, fungi are an overlooked symbiont of nematode microbiota. Several studies on plant parasitic nematodes have documented numerous associated fungi species spanning from saprophytics, plant pathogens, root endophytes, and nematode pathogens. Members of Ascomycota have been the most abundant and frequently associated with *Heterodera* and *Globodera* nematodes, with *Fusarium* being the representative fungal genera (Hu et al. 2020; Jumbam et al. 2024). The function of fungi in nematode biology is unknown, and most studies have focused on external fungal species attached to nematode cuticles. Some authors proposed that saprotrophic fungi can colonize *Heterodera* eggs within cysts and protect them from parasitic fungi (Chen and Chen 2002). Thus, fungi–nematode symbiosis deserves more attention, not only in plant‐parasitic nematodes but in all nematode functional groups. Considering fungivorous nematodes and that fungi are part of the diet of numerous nematodes, commensal/mutualist associations among them are expected to occur, as seen in bacterivore nematodes.

RNA viruses are assumed to inhabit every cellular life form (Koonin et al. 2006). Despite their ubiquity, knowledge about RNA viruses comes from medical and economically important diseases, representing only a tiny fraction of the real virus diversity. Metagenomic studies of invertebrate viruses are scarce but have revealed a greater viral diversity than those of vertebrates. In nematodes, RNA viruses have been documented in plant‐parasitic (Bekal et al. 2014; Frézal et al. 2019; Vieira and Nemchinov 2019), animal‐parasitic (Quek et al. 2024; Williams et al. 2019), and free‐living species (Félix and Wang 2025; Vieira et al. 2022). By screening published transcriptomes, Quek et al. (2024) discovered 75 RNA viruses associated with 27 animal‐parasitic nematodes (e.g., *Ascaris lumbricoides*, *Haemonchus contortus*, *Trichinella spiralis*, *O. volvulus*, *B. malayi*). The extensive diversity and conserved global spread of nematode‐virus associations, such as those of *A. sum*, *A. lumbricoides*, and *O. volvulus*, suggest an ancient and stable co‐evolution. The full extent and diversity of nematode RNA viruses, their role in nematode biology, and their role as drivers or modulators of pathogenesis (by parasitic nematodes) are poorly explored. However, some evidence would enlighten some hypotheses about nematode‐virus interactions. For example, RNA viruses inhabiting the protozoan parasites *Leishmania* sp. or *Trichinella* sp. (Leishmania virus 1 and Trichinella virus 1) induce hyperinflammatory immunity, driving disease pathogenesis and subverting host immunity (Fichorova et al. 2012; Ives et al. 2011). Indeed, the BMRV1 and OVRV1 viruses from *B. malayi* and *O. volvulus*, respectively, elicited antibody responses from host immune cells, suggesting a potential modulation of host immunity (Quek et al. 2024). Given that viruses have been involved in key biological phenomena like placental origin (Chuong 2018) or the evolution of braconid wasp parasitoidism (Quicke and Butcher 2021), they are expected to provide valuable contributions to Nematoda's evolutionary history (Jones et al. 2005). Unveiling how viruses interact with nematodes will enrich our comprehension of nematodes' natural history, with the associated microbiome (e.g., bacteria, fungi, viruses) being a crucial promoter.

Despite their potential ecological relevance, nematode–protozoa interactions remain poorly understood. In natural environments, nematodes have been reported to feed on protozoa (Yeates 1987). Members of the families Diplogasteridae and Mononchidae, for instance, are bacterivorous during their larval stages but become predators of both nematodes and protozoa as adults (Yeates et al. 1993; see also Rønn et al. 2012 for review). These interactions appear to be more than incidental. For example, *Mesodiplogaster* individuals feeding on amoebae exhibit faster growth rates than those consuming only bacteria (Elliott et al. 1980), suggesting that protozoa may offer enhanced nutritional benefits. Conversely, some protozoa, particularly large members of the Vampyrellidae, prey on nematodes (Hess et al. 2012). Species such as *Vampyrella lateritia* create perforations in nematode cells to extract cytoplasm, while others, like *Arachnula impatiens* and *Theratromyxa weberi*, engulf their prey via phagocytosis (Gong et al. 2015). However, most evidence for protozoan predation on nematodes comes from laboratory studies, and more field‐based research is needed to understand the ecological relevance and dynamics of these interactions (Rønn et al. 2012). Finally, it has been documented protozoan ciliates as epibionts on marine nematodes (Baldrighi et al. 2020). But the potential costs and benefits of this interaction has not been established.

## 9 Conclusions and Future Directions

**FIGURE 3:**
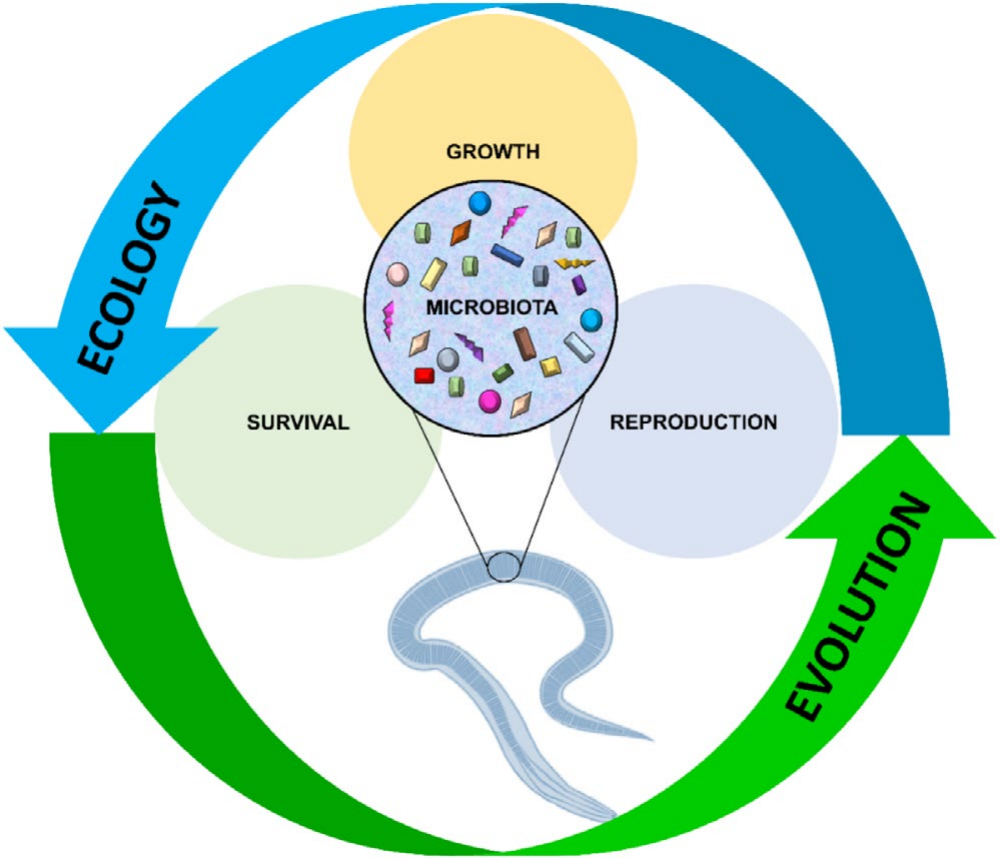
The microbiota, including viruses, fungi, and bacteria, can influence nematode life‐history traits, with direct consequences for their ecology and evolutionary dynamics.

Microbiota is an essential component of every organism as it supports diverse biological processes. Either complementing metabolic pathways, synthesizing nutrients, or countering environmental threats, microbiota stands as a stepping‐stone that favors nematodes’ free‐living or parasitic lifestyles. There is a solid framework about the most recognized nematode‐bacteria complexes, with a notable comprehension of the associated core symbionts. However, a review of current research underscores the limited understanding of microbiota functions within *C. elegans* and even more so in other nematode species. Elucidating these functions is essential for understanding how microbiota maintains internal functional balance and how it responds to environmental perturbations. Identifying the ecological conditions that favor specific bacterial assembly will not only improve our understanding of host–microbiota mutualisms but also shed light on bacterial colonization strategies. Furthermore, this line of research is likely to uncover novel bacterial metabolic capabilities with potential applications in health, agriculture, and biotechnology. It is worth mentioning that fungi, viruses, and protozoans are commonly ignored components of microbiota. Nonetheless, emerging evidence suggests an essential role of these members in nematodes. Thus, including fungi, viruses, and protozoans will improve our comprehension of nematode–microbiota outcomes from a holistic perspective. Depending on microbiota composition, nematode phenotypes may vary considerably with implications at both ecological and evolutionary scales. By studying nematode life‐history traits, we can assess the evolutionary outcomes of microbiota effects (Figure 3). Applying life‐history theory to microbiota research would reveal how different microbiotas promote phenotypic variation and evolutionary trade‐offs, which in turn lead to adaptation. However, comparative studies across diverse nematode and microbiota types are needed to quantify the extent and limitations of these effects. We propose the application of the life‐history framework to better understand how resources are distributed among host fitness components and microbial partners. This theory can help clarify the physiological basis of trade‐offs between life‐history traits and the role of microbiota in host adaptation. It is also critical to distinguish the direct effects of microbiota from those attributable to bacterial nutritional value. Identifying which and how bacteria are digested versus retained within the gut will help resolve the mechanisms underlying observed phenotypic changes.

In addition, studies should focus on microbiota that closely resemble those found in natural environments. Tools such as metabolic pathway modeling and physiological profiling of individual bacterial strains can help determine how microbial diversity influences host traits. For example, phenomic microarrays (e.g., Biolog) can assess the metabolic capacities of bacteria selected from metagenomic datasets. At the community level, metabolic network modeling can elucidate interactions among coexisting bacterial taxa and their functional contributions to the host. Expanding this research beyond *C. elegans* to other nematodes offers promising opportunities to deepen our understanding of microbiota evolution and ecology across the phylum. Several emerging technologies now facilitate high‐resolution investigation of host–microbiota interactions. These include bacterial biosensors that detect biochemical changes in the gut, bioluminescent bacteria for visualizing colonization dynamics, and optogenetic tools that modulate bacterial metabolism and indirectly influence host physiology. Collectively, these advances promise to illuminate previously unexplored aspects of microbial symbiosis in nematodes and metazoans more broadly.

## Author Contributions

**Víctor José Trejo‐Meléndez:** conceptualization (lead), visualization (equal), writing – original draft (lead), writing – review and editing (lead). **Jorge Contreras‐Garduño:** funding acquisition (lead), supervision (lead), validation (lead), writing – review and editing (supporting).

## Conflicts of Interest

The authors declare no conflicts of interest.

## Data Availability

The authors have nothing to report.
